# The iNs and Outs of Direct Reprogramming to Induced Neurons

**DOI:** 10.3389/fgeed.2020.00007

**Published:** 2020-09-04

**Authors:** Jasmine L. Carter, Julian A. N. M. Halmai, Kyle D. Fink

**Affiliations:** Gene Therapy Center, Stem Cell Program, Department of Neurology, Institute for Regenerative Cures, University of California, Davis, Sacramento, CA, United States

**Keywords:** induced neuron, direct reprogramming, stem cells, CRISPRa, dCas9, epigenetic editing

## Abstract

Understanding of cell-type specific transcription factors has promoted progress in methods for cellular reprogramming, such as directly reprogramming somatic cells to induced neurons (iN). Methods for direct reprogramming require neuronal-fate determining gene activation via neuron-specific microRNAs, chemical modulation of key neuronal signaling pathways or overexpression via viral vectors, with some reprogramming strategies requiring a combination of these methods to induce the neuronal-cell fate. These methods have been employed in a multitude of cell types, including fibroblasts, hepatocytes, peripheral blood mononuclear, and T cells. The ability to create iN from skin biopsies and blood samples coupled with recent advancements in artificially inducing age- and disease-associated phenotypes are accelerating the development of disease models for late-onset neurodegenerative disorders. Here, we review how activation of the neuronal transcriptome alters the epigenetic landscape of the donor cell to facilitate reprogramming to neurons. We also discuss the advantages of using DNA binding domains such as CRISPR/dCas9 to overcome epigenetic barriers to induce neuronal-cell fate by activating endogenous neuronal cell-fate determining genes.

## Introduction

**Figure 1:**
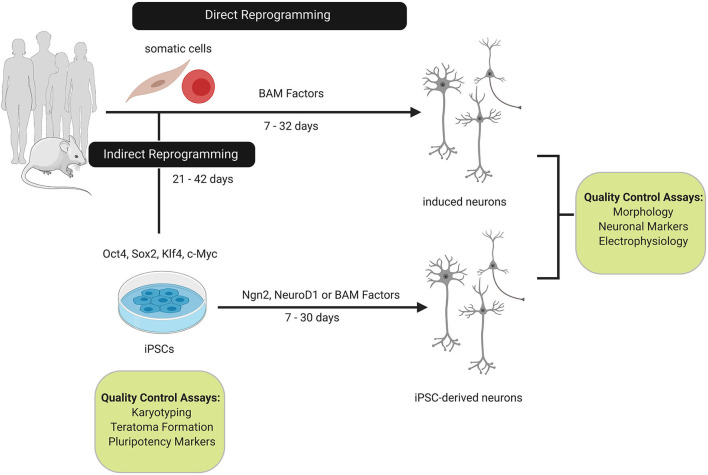
Comparison of direct and indirect reprogramming to the neuronal cell-fate. Somatic cells from humans or mice can be directly or indirectly reprogrammed to neurons. Direct reprogramming can be achieved by overexpressing Brn2, Ascl1, and Myt1L (BAM factors) which reprogram somatic cells within 32 days by activating neuronal networks involved in neurogenesis and neuronal differentiation. In contrast, somatic cells can be reprogrammed to induced pluripotent stem cells (iPSCs) with the Yamanaka factors (Oct4, Sox2, c-Myc, and Klf4). Reprogramming to the intermediate iPSC state extends the reprogramming timeline as quality control assays for karyotypic stability and pluripotency must be conducted. Overexpression of the BAM factors or supplemental neurogenesis factors, such as, Ngn2 or NeuroD1 are sufficient to differentiate iPSCs to neurons. Induced and IPSC-derived neurons are characterized based on morphology, pan-neuronal and subtype markers, and functional properties.

The ability to model human disease *in vitro* was transformed by the reprogramming of somatic cells into induced pluripotent stem cells (iPSCs) with defined factors (Takahashi and Yamanaka, 2006; Takahashi et al., 2007). This breakthrough was followed by the differentiation of iPSCs into the neuronal lineage which outlined master regulators and signal transduction pathways involved in establishing a neuronal phenotype *in vitro* (Figure 1) (Chambers et al., 2009; Karumbayaram et al., 2009; Cooper et al., 2010). Differentiating iPSCs to the neuronal-cell fate uncovered diverse neuronal networks associated with reprogramming and has benefited disease modeling efforts for neurodegenerative disorders (Dimos et al., 2008; Park et al., 2008; Chambers et al., 2009; Soldner et al., 2009; Marchetto et al., 2010; Urbach et al., 2010; Yagi et al., 2011). iPSC-derived neurons have become an essential tool for interrogating neuronal function and developing gene or pharmaceutical therapies, yet there are limitations that need to be understood when studying the underlying molecular mechanisms of neurodegenerative disorders (Egawa et al., 2012; Pei et al., 2016; Kondo et al., 2017; Wang et al., 2017).

Multiple groups have reported a failure for diseased and control iPSC-derived neurons to mature beyond fetal development milestones determined via whole transcriptome analysis with RNA sequencing (RNA-seq). These iPSC-derived neurons cluster with RNA samples derived from the murine proliferative germinal zone (Lim et al., 2017; Mehta et al., 2018). In line with the finding that iPSC-derived neurons fail to mature, Huntington's Disease and control iPSC-derived neurons share similar expression profiles to the fetal cerebral cortex after 130 days in culture, thus creating a bottleneck for efficiently generating mature neuronal models (Mehta et al., 2018). Reprogramming to a pluripotent state has also been shown to alter levels of oxidative stress, DNA damage, DNA methylation, and telomere length evident in patient-derived fibroblasts which erases age-phenotypes relevant to disease progression (Maherali et al., 2007; Saha and Jaenisch, 2009; Lapasset et al., 2011; Yagi et al., 2011; Huh et al., 2016). Moreover, constitutive expression of pluripotent reprogramming factors can lead to karyotypic instability and prevents differentiation of iPSCs to the neuronal lineage (Ramos-Mejia et al., 2010; Ramos-Mejía et al., 2012). These findings present a potential limitation for iPSC-derived neurons to mature and exhibit key pathologies of age-related neurodegenerative disorders, while highlighting the importance of establishing tools for neuronal reprogramming which delineate developmental and pathological milestones *in vitro*.

Developments in the cellular reprogramming field have enabled somatic cells to be directly reprogrammed to induced neurons (iN) (Vierbuchen et al., 2010). These iN maintain epigenetic signatures of the donor cell and have now become a source for studying the underlying molecular mechanisms of late-onset neurodegenerative disorders (Hu et al., 2015; Huh et al., 2016; Liu et al., 2016; Abernathy et al., 2017). Here, we discuss traditional and novel methods for direct reprogramming to the neuronal cell-fate and explore how progress in reprogramming techniques enables disease- and age-associated phenotypes to be recapitulated *in vitro*. In this review we will focus on (1) transcription factor-based strategies which use neurogenesis and neuronal subtype factors to induce reprogramming, (2) microRNA-based repression of competing donor-cell fates, and (3) the utility of small molecule-based reprogramming to activate neurogenic signal transduction pathways.

## Transcription Factor Interactions During Direct Reprogramming

Direct reprogramming to neuronal cells requires activation of neuronal-fate determining genes, chemical modulation of key neuronal signaling pathways, overexpression via viral vectors, or endogenous activation using DNA binding domains (Table 1). Many groups have reported specific neuronal fate determining genes promoting reprogramming through neurogenic pathway activation and subsequent donor cell identity repression. Gene expression analysis during neurogenesis highlighted the importance of the transcription factors *Brn2, Ascl1, Myt1l* (*BAM* factors), and *NeuroD1*. These transcription factors regulate gene expression networks which establish and specify the neural identity (Vierbuchen et al., 2010; Pang et al., 2011). The role of *NeuroD1* in adult neurogenesis was uncovered by ablation in the hippocampus and lateral ventricles, which prevented neural progenitor cells (NPCs) from differentiating and maturing in mice (Gao et al., 2009). *NeuroD1* ablation also resulted in increased neuronal cell death *in vitro* and *in vivo*. Interestingly, it has been reported that *NeuroD1* alters the epigenetic context of several genes such as *Hes6, Brn2*, and *Sox1*. ChIP-qPCR analysis revealed the chromatin structure and accessibility become susceptible to reprogramming as NeuroD1 interacts with the regulatory elements of genes related to neurogenesis, suggesting NeuroD1 is a master regulator which induces the neuronal cell-fate. However, this study differentiated embryonic stem cells (ESCs) to pyramidal neurons rather than employing direct reprogramming strategies to track NeuroD1 activity, which potentially differs when the starting cell is pluripotent (Pataskar et al., 2016). Ectopic NeuroD1 expression is also sufficient to reprogram reactive glial cells to induced neurons *in vivo* (Guo Z. et al., 2014). This study showed how resident cells in a complex organ system can be directly reprogrammed to glutamatergic and GABAergic neurons with a single transcription factor. These findings build on previous reports which describe a molecular mechanism whereby transcription factors such as, Ascl1 and Ngn2 reprogram astrocytes to functional iN which fire action potentials and exhibit altered Na^+^ currents following tetrodotoxin (TTX) treatment (Berninger et al., 2007). Indeed, confirming *in vivo* reprogramming of NG2 glia to iN with rabies virus tracing indicates integration of iN into host circuitry is feasible and dependent on the reprogramming factors (Torper et al., 2015).

**Table 1:** Summary of strategies for direct reprogramming to induced neurons.

**References**	**Donor cell**	**Target cell**	**Methods and reprogramming factors**	**Characterization**			
				**FACS or ICC**	**Functional**	**Neurotransmitter**	**Epigenetic characterization**
Pataskar et al. (2016)	Mouse embryonic stem cells	Pyramidal neurons	Viral vector; NeuroD1	Yes; Tuj1	ND	ND	ChIP-seq, ChIP-qPCR, and RNA-seq
Vierbuchen et al. (2010)	Mouse embryonic and postnatal fibroblasts	Neurons	Viral vector; Ascl1, Brn2, Myt1L	Yes; Tuj1, MAP2, NeuN, and Synapsin	Patch-clamp: voltage clamp and current clamp	vGLUT1 and GABA	ND
Chanda et al. (2014)	Human fetal and postnatal fibroblasts	Neurons	Viral vector; Ascl1	ND; Tuj1, NeuN, and Synapsin	Patch-clamp: voltage clamp and current clamp	vGLUT1	Fluidigm biomark
Ring et al. (2012)	Mouse embryonic fibroblasts and human fetal fibroblasts	Neural stem cells	Viral vector; Sox2	ND; Sox2, Nestin, Pax6, BLBP, Tuj1, MAP2, GFAP, O4, Olig2	Patch-clamp: voltage clamp and current clamp	vGLUT1 and GABA	Bisulfite sequencing and Microarray
Han et al. (2012)	Mouse fibroblasts	Neural stem cells	Viral vector; Brn2, Sox2, Klf4, Tcf3, and +/– c-Myc	ND; SSEA1, Olig2, GFAP, Tuj1, O4, Sox2, Nestin, Ascl1, Ng2, and S100B	Patch-clamp: voltage clamp and current clamp	vGLUT1, GABA, TH	Bisulfite sequencing and Microarray
Marro et al. (2011)	Mouse hepatocytes	Neurons	Viral vector; Ascl1, Brn2, and Myt1L	Yes; PSA-NCAM, MAP2, NeuN, and Synapsin	Patch-clamp: voltage clamp and current clamp	vGLUT1, GABA, TH	Microarray
Tanabe et al. (2018)	Human peripheral blood mononuclear and T cells	Neurons	Viral vector; Ascl1, Brn2, Myt1L, and Ngn2	Yes; Tuj1, MAP2, NeuN, and Synapsin, SATB2 and CTIP2	Patch-clamp: voltage clamp and current clamp	vGLUT1	RNA-seq
Pang et al. (2011)	Human fetal and postnatal fibroblasts	Neurons	Viral vector; Ascl1, Brn2, Myt1L, and NeuroD1	ND; Tuj1, MAP2, NeuN, PSA-NCAM, Synapsin, Tbr1, and Peripherin	Patch-clamp: voltage clamp and current clamp	vGLUT1, VGLUT2, GABA, and TH	Fluidigm dynamic array
Yoo et al. (2011)	Human neonatal and adult fibroblasts	Neurons	micro RNA; miRNA 9/9* and miRNA 124	Yes; Tuj1, MAP2, Synapsin, Neurofilament, Pax6, Sox2, Tbr2, SCN1A, and NMDAR1	Patch-clamp: voltage clamp and current clamp	vGLUT1, GAD67	Fluidigm biomark
Li et al. (2015)	Mouse fibroblasts	Neurons	Chemical modulation; Forskolin, ISX9, CHIR99021, and SB431542	ND; Tuj1, NeuN	Patch-clamp: voltage clamp and current clamp	vGLUT1 and GABA	RNA-seq
Abernathy et al. (2017)	Human adult fibroblasts	Motor neurons	micro RNA and viral vector; miRNA 9/9*, miRNA 124, ISL1, and LHX3	ND; Tuj1, MAP2, NeuN, SCN1A, Ankyrin G, SV2, NCAM, MNX1, CHAT, SMI-32	Patch-clamp: voltage clamp and current clamp	MNX1, CHAT, SMI-32	ChIP-seq, ATAC-seq, MeDIP-seq, MRE-seq, RNA-seq, and Microarray
Herdy et al. (2019)	Human fibroblasts	Neurons	Chemical modulation; ZM336372, Pyrintegrin, AZ960 and KC7F2	Yes; Tuj1, NeuN, PSA-NCAM	Calcium imaging	vGLUT1 and GABA	MethylationEPIC BeadChip and RNA-seq
Hu et al. (2015)	Human fibroblasts	Neurons	Chemical modulation; Valproic acid, CHIR99021, Repsox, forskolin, SP600125, GO6983, Y-27632	ND; Tuj1, MAP2, Dcx, Tau, NeuN, Synapsin	Patch-clamp: voltage clamp and current clamp and Calcium imaging	vGLUT1	Fluidigm biomark
Shahbazi et al. (2016)	Mouse embryonic fibroblasts, human neonatal, fetal, and adult fibroblasts	Neural stem cells	Artificial transcriptional activator; Zfp521	ND; Tuj1, Nestin, Sox1, SOX2, GFAP, PAX6, NCAM, CD133, OTX2, EMX1, HOXA2, HOXB2, NKX6.1, Synapsin, MAP2, O4	Patch-clamp: voltage clamp and current clamp	vGLUT1, GABA, CHAT, HB9, DAT, and TH	RNA-seq
Black et al. (2016)	Mouse embryonic fibroblasts	Neurons	Artificial transcriptional activator (VP64-dCas9-VP64 and sgRNAs); Ascl1, Brn2, and Myt1l	ND; Tuj1, MAP2	Patch-clamp: voltage clamp and current clamp	ND	ChIP-seq and ChIP-qPCR
Liu Y. et al. (2018)	Mouse embryonic stem cells	Neurons	Artificial transcriptional activator (dCas9-SunTag and sgRNAs); Ascl1 or Ngn2	Yes; Tuj1, MAP2, NeuN, Synapsin, PSA-NCAM, GLT1, Olig2, Sox10	Patch-clamp: voltage clamp and current clamp	GAD65	RNA-Seq
Baumann et al. (2019)	Mouse neural progenitor cells	Neural stem cells	Artificial transcriptional activator(dCas9-TET1 and dCas9-VP64 and sgRNAs); Sox1	Yes; Tuj1, GFAP, Sox1, Ocln, Zo-1, Nestin, Notch1	ND	vGlut1 and Calbindin	ChIP-seq, Bisulfite sequencing; and RNA-seq

**Figure 2:**
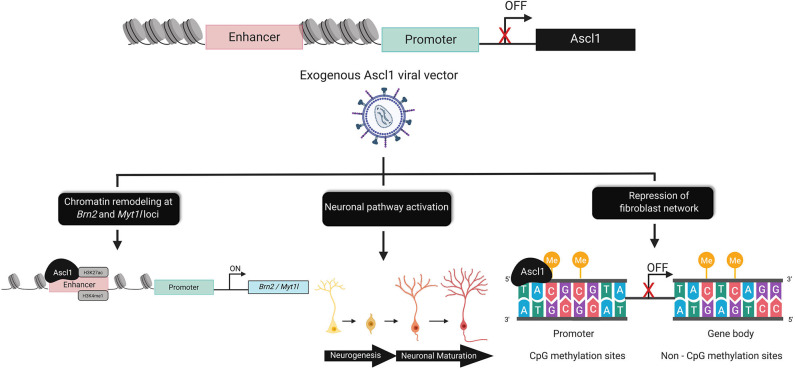
Epigenomic alterations underlying direct reprogramming. The chromatin dynamics of Ascl1, prevent expression of the on-target pioneer transcription factor in fibroblasts. Exogenous Ascl1 binds to target loci, inducing chromatin remodeling at regions enriched for activating histone marks such as, H3K27ac and H3Kme1. Pathways associated with neurogenesis and neuronal maturation are directly and indirectly activated by Ascl1. Ascl1 facilitates global DNA-methylation changes that are sufficient to rewrite the epigenetic memory of the donor cell by increasing CpG methylation at the promoter and non-CpG (CpA, CpT, and CpC) sites within the gene body of fibroblast genes.

A comprehensive study analyzed 19 genes related to neuronal differentiation and nervous tissue development, and identified the BAM factors as being sufficient to directly convert mouse embryonic and postnatal fibroblasts into neuronal cells (Vierbuchen et al., 2010). Importantly, subsequent characterization during murine neurodevelopment demonstrated that neurogenesis *in vivo* is regulated by Ascl1 (Castro et al., 2011). Reprogramming studies using single factor induction with Ascl1 show endogenous *Brn2* and *Myt1l* loci undergo significant chromatin remodeling 5 days post-induction, suggesting that Ascl1 promotes accessibility of genes involved in neuronal maturation (Wapinski et al., 2013, 2017; Raposo et al., 2015). Interestingly, comparison to primary neurons revealed enrichment for H3K27ac and DNase-I hypersensitivity at endogenous *Brn2* (Wapinski et al., 2017). These findings suggest a single transcription factor is sufficient to induce reprogramming by facilitating chromatin remodeling and neuronal pathway activation. Analysis of global changes in DNA methylation during direct reprogramming to neurons reveals that reprogramming with Ascl1 alone resulted in CpG methylation at promoters of fibroblast genes and promoted methylation at non-CpG (CpA, CpT, and CpC) regions. Large-scale changes to non-CpG methylation sites within gene bodies was observed following combined expression of the BAM factors. These signatures are similar to the patterns observed in mature cortical neurons, which is consistent with reports of Brn2 and Myt1l role in neuronal maturation (Chanda et al., 2014; Luo et al., 2019). Methylation at non-CpG sites following direct reprogramming suggests a key developmental signature is recapitulated in iN as this signature is a hallmark of mouse and human neurodevelopment (Varley et al., 2013; Guo J.U. et al., 2014) (Figure 2).

However, in contrast to mouse studies, BAM alone was not sufficient to reprogram human fetal fibroblasts and required the addition of NEUROD1 (Pang et al., 2011). BRN2 recruitment to genomic sites contributes to maturation and neurite extension following the initiation of iN reprogramming (Wapinski et al., 2013). The reprogramming process is further supported by MYT1L which maintains neuronal identity by repressing non-neuronal differentiation programs (Wapinski et al., 2013; Treutlein et al., 2016; Mall et al., 2017). Interestingly, ectopic expression of *ASCL1* alone is sufficient to convert human fetal and postnatal fibroblasts to neurons, albeit converted cells express immature neuronal surface markers and exhibit immature morphology (Chanda et al., 2014). ASCL1 has been characterized as a pioneer transcription factor that can bind to target sites in human fibroblasts, MEFs and pluripotent-derived NPCs (Castro and Guillemot, 2011; Wapinski et al., 2013; Raposo et al., 2015). These findings support the master regulator activity of ASCL1 in human neuronal reprogramming.

Direct reprogramming of mouse and human fibroblasts to lineage-restricted neural stem cells (NSCs) has been explored with the transcription factor Sox2, coupled with EGF and FGF2 growth factors (Lujan et al., 2012; Ring et al., 2012). However, analysis of NSCs derived from ectopic expression of *Brn4, Sox2, Klf4, Tcf3*, and with or without *c-Myc*, suggests that the fibroblast cell-fate network is still active in late passage NSC cultures (Han et al., 2012). Interestingly, the neural stem cell marker Nestin, displays differential DNA methylation in NSCs and fibroblasts, with the second intron of the loci being unmethylated in NSCs (Dong et al., 2009). This was evident in directly reprogrammed NSCs, suggesting direct reprogramming to the NSC state retains key DNA methylation signatures (Han et al., 2012). It becomes evident that understanding the epigenetic signature upon induction of the neuronal transcriptome is critical in order to predict the outcome of the reprogramming process (Han et al., 2012).

## Induced Neurons Derived From Mesodermal and Endodermal-Derived Cells

Canonical reprogramming strategies have focused on developing iNs from donor cells of the ectoderm, such as mouse embryonic, tail-tip fibroblasts or human neonatal, and adult fibroblasts (Maherali et al., 2007; Vierbuchen et al., 2010; Pang et al., 2011; Yoo et al., 2011; Torper et al., 2013; Wapinski et al., 2013, 2017; Chanda et al., 2014; Raposo et al., 2015; Huh et al., 2016; Abernathy et al., 2017). However, a study co-expressed the BAM factors in primary mouse hepatocytes to further investigate the feasibility of reprogramming cells derived from the endodermal lineage to iN (Marro et al., 2011). The transcriptomic analysis performed in this study suggests that reprogramming across germ layers, or at least to a neuronal fate, is more challenging than fibroblasts to neurons. Hepatocyte derived-iN displayed neuronal morphology and generated spontaneous action potentials. Single-cell transcriptomic analysis of primary neurons and hepatocyte-derived iN revealed similar expression of liver markers in hepatocyte-derived iN, suggesting these markers are transcriptional noise. The consequence of retaining epigenetic signatures or expression profiles from a different germ layer has yet to be analyzed in hepatocyte-derived iN. In order for iN to provide accurate modeling systems for therapeutic drug discovery, it is critical for the donor cell's transcriptional network to be silenced or the impact retention has on neuronal function must be investigated. These findings confirm the potential for fibroblasts to be the gold standard for inducing the neuronal-cell fate as they can be efficiently obtained and resemble a similar molecular profile useful for disease modeling.

In line with developing disease models, peripheral blood mononuclear cells can be obtained in blood samples with an efficient medical procedure and thereby have the potential to expand patient-specific disease modeling efforts. Recently, peripheral blood mononuclear (PBMC) as well as T-cells were reprogrammed to iN with the BAM factors and Ngn2, a transcription factor involved in coordinating chromatin accessibility during neurogenesis (Tanabe et al., 2018; Aydin et al., 2019). Previous fibroblast-derived iN studies showed that supplementing the reprogramming medium with growth factors and glial monolayers increased reprogramming efficiency (Vierbuchen et al., 2010; Pang et al., 2011). It has also been reported that astrocyte co-cultures eliminate variability of functional maturity in iN (Bardy et al., 2016). Interestingly, improved reprogramming efficiency of PBMCs occurred following the addition of small molecules targeting pathways involved in neural induction during development, such as BMP and TGF-B pathway inhibition and PKA activation (Tanabe et al., 2018). PBMCs transfected with the BAM factors and Ngn2 were seeded on primary mouse glial cells. Interestingly, 5 days post-transfection the viability of primary mouse glia decreased, suggesting the potential for donor cells in the early stages of reprogramming to retain an immunogenic memory (Tanabe et al., 2018). These findings highlight the possibility that donor cell identity can dictate the optimal condition requirements to improve reprogramming efficiency. Transcriptome analysis in this study demonstrated activation of the neuronal network through enrichment of genes associated with neural development, synaptic transmission, and pan-neuronal markers while genes associated with PBMCs were downregulated. While creating iN from mesodermally-derived cells is innovative, the exact mechanisms underlying transcription factor and small molecules reprogramming at the epigenetic level are unclear in this model. Parallel comparisons of iN derived from PBMCs, specialized lymphocyte populations and fibroblasts may also lead to identification of optimal reprogramming transcription factors, small molecules, microRNAs, or substrates for each donor cell type.

## Small Molecules Induce A Distinct Neuronal Network During Reprogramming

Human adult fibroblasts can also be directly converted to iN with high conversion efficiencies by lentiviral induction of neuronal microRNAs (miRNA) and transcription factors (Pang et al., 2011; Yoo et al., 2011; Abernathy et al., 2017). Here, miRNA-9/9^*^ and miRNA-124 facilitate reprogramming by repressing the neuronal-cell fate inhibitor RE1-silencing transcription factor (REST) (Yoo et al., 2011; Lu and Yoo, 2018). The transcriptome of these iN suggests 30 days of miRNA-9/9^*^ and miRNA-124 activity was necessary to fully reprogram fibroblasts to mature iN even though significant changes to fibroblast and neuronal gene regulation occurred within 10 days of miRNA expression. RNA-seq analysis of miRNA-iN showed an increase in levels of transcripts related to neuronal projections, neurotransmission and synapses, and correlated with electrophysiological properties of functional neurons *in vitro* (Abernathy et al., 2017). Interestingly, additional transcriptome analysis revealed changes in the expression of genes associated with modulating the epigenetic landscape. These genes included the DNA-methyltransferase, *DNMT3A*, and chromatin remodeling proteins such as *CHD5* and *CHD7*. miRNA-mediated reprogramming did not induce transcriptional changes for ASCL1, a finding that was also observed following miRNA-mediated reprogramming of mouse fibroblasts (Yoo et al., 2011; Li et al., 2015; Abernathy et al., 2017). While ASCL1 is a master regulator of neurogenesis, these findings suggest that distinct neuronal networks can generate the neuronal-cell fate. Moreover, small molecules, such as, Forskolin, ISX9, CHIR99021, and SB431542 (FICS) that recapitulate the Ascl1 network in mouse fibroblasts, are sufficient to initiate neuronal reprogramming, downregulate fibroblast network genes and promote the development of neurons with complex morphologies (Li et al., 2015). FICS-iN appeared to benefit from the brain microenvironment being recapitulated as co-culture with primary astrocytes or neurons resulted in a significant increase in functional properties (Li et al., 2015). The findings observed with FICS-iN co-cultures parallel a previous report which showed accelerated functional properties of neuroepithelial cells corresponded to astrocyte differentiation and maturation in co-cultures (Johnson et al., 2007). These neuronal and astrocyte co-culture studies highlight the importance of identifying the optimal *in vitro* conditions to create functional iN models.

Recently, human fibroblasts from aged donors were reprogrammed to iN following doxycycline treatment with an inducible Tet-On construct for *NGN2* and *ASCL1* (Herdy et al., 2019). The expression construct drives tetracycline expression from the UbC promoter while *NGN2* and *ASCL1* are under the TREtight promoter, thus creating the all in one UNA construct. When compared to individual reprogramming vectors for ASCL1 and NGN2, the UNA construct successfully increased the number of NeuN or Tuj1 positive iN and reprogramming efficiency by >90%. To further characterize UNA-mediated iN, RNA-seq analysis was used to identify pathways enriched during a time-course transcriptome analysis of reprogrammed iN. Small molecule screening was then performed to identify activators or inhibitors which could modulate the 10 top pathways and replace the UNA construct. Four small molecules were identified, ZM336372, Pyrintegrin, AZ960, and KC7F2 (ZPAK) that increased the number of iN by activating signaling cascades associated with cell cycle regulation, cytoskeletal formation and organization, and metabolic regulation. Analysis of global DNA methylation in ZPAK iN derived from young and aged fibroblasts revealed a retention of age-dependent CpG methylation when compared to the donor fibroblast (Herdy et al., 2019). Such studies in iN derived from young and aged fibroblasts are crucial to assess if age-associated signatures are retained following reprogramming (Yoo et al., 2011; Huh et al., 2016; Luo et al., 2019).

## Modeling Late-Onset Neurodegenerative Disorders With Induced Neurons

The advent of *in vitro* reprogramming technologies have enabled disease-in-a-dish models for complex neurodegenerative disorders to be developed and accelerated therapeutic pipelines to test novel compounds. However, identifying the optimal reprogramming technique can be challenging and dependent on underlying mechanisms associated with disease pathology. Small molecules can activate neuronal signaling pathways and facilitate reprogramming without requiring exogenous factors. Alzheimer's disease (AD) fibroblasts containing familial mutations in *APP* and *presenilin 1* were reprogrammed to iN using a 7-factor small molecule cocktail. This cocktail was modified from a minimal cocktail (Valproic acid, CHIR99021, Repsox: VCR) sufficient to differentiate NPCs to neurons by adding forskolin, SP600125, GO6983, Y-27632 (VCRFSGY). While VCRFSGY reprogrammed fibroblasts to Doublecortin (Dcx), Tuj1, and Map2 positive cells, additional molecules were necessary to improve maturation and survival 7 days post-chemical reprogramming. Culturing iN with CHIR99021, Forskolin, Dorsomorphin, BDNF, GDNF, and NT3 for an additional 2 weeks created functionally mature iN capable of generating evoked action potentials. Importantly, VCRFSGY was successful in reducing the fibroblast-cell fate, thereby minimizing the potential for the donor cell network to influence gene expression network interactions in a disease model. Alzheimer's patient-derived fibroblasts reprogrammed to iN with VCRFSGY revealed higher amyloid beta (Aß) production compared to control iN (Hu et al., 2015). These findings are consistent with previous reports of elevated Aß production in AD iPSC-derived neural progenitors and neurons (Israel et al., 2012; Choi et al., 2014; Muratore et al., 2014; Raja et al., 2016). Moreover, chemical modulation with VCRFSGY suggests reprogramming can be optimized to decrease the donor cell identity and retain disease-associated phenotypes in mature neurons (Hu et al., 2015).

Small molecules have also been used to reprogram patient-derived fibroblasts to specific subtypes of neurons, a particularly advantageous tool for modeling complex neurodegenerative disorders at the cellular level. A human induced-motor neuron (iMN) disease model for Amyotrophic lateral sclerosis (ALS) provided insightful information on studying disease-associated phenotypes *in vitro*. These studies enabled evaluation of the safety and efficacy of the small molecule GSK-3 inhibitor, kenpaullone (Yang et al., 2013; Liu et al., 2016). Here, >86% reprogramming efficiency was achieved by chemical modulation with forskolin, dorsomorphin, and basic fibroblast growth factor paired with *NGN2, SOX11, ISL1*, and *LHX3*. Interestingly, this reprogramming strategy bypassed the neural progenitor state and benefited from mouse astrocytes co-cultures which improved the complexity and survival of iMN cultures. Further characterization of these iMN revealed an ability to form functional neuromuscular junctions and the expression of cholinergic markers. ALS-derived iMNs displayed traditional disease relevant phenotypes, such as, cytoplasmic FUS accumulation, reduced soma size, and altered synaptic activity. Kenpaullone successfully rescued ALS disease-associated phenotypes; however, removal of kenpaullone reversed its effects, suggesting that the iMNs became dependent on the small molecule drug (Liu et al., 2016). These findings suggest therapeutic screening for neurodegenerative disorders can be improved by optimizing methods to reprogram patient-derived fibroblasts to specific neuronal subtypes.

Multiple strategies have been employed to create dopaminergic neuronal models for Parkinson's disease (PD) (Caiazzo et al., 2011; Pfisterer et al., 2011; Theka et al., 2013; Jiang et al., 2015). Pairing the BAM factors with dopaminergic factors, such as Lmx1a and FoxA2, is sufficient to directly reprogram human fibroblasts to induced dopaminergic (iDA) neurons that express tyrosine hydroxylase and markers associated with dopamine synthesis (Pfisterer et al., 2011). A similar approach was employed by reprogramming adult mouse fibroblasts to a dopaminergic fate with Ascl1, Nurr1, and Lmx1a (Caiazzo et al., 2011). Here, the promoter of tyrosine hydroxylase and vesicular monoamine transporter 2 (Vmat2) was unmethylated which suggests specification factors induce mechanisms which alter the methylation signature of genes involved in dopamine synthesis. Characterization of iDA revealed the presence of functional D2 receptors and dopamine release following KCl stimulation, thereby supporting the potential for direct reprogramming to yield functional neuronal subtypes. Moreover, establishing PD disease models is possible as Ascl1, Nurr1, and Lmx1a successfully reprogram healthy and PD patient-derived fibroblasts to iDA. A subsequent study used Ascl1, Nurr1, and Lmx1a with miRNA124 and p53 knockdown (ANLmp) to reprogram fibroblasts to iDA (Jiang et al., 2015). Interestingly, these reprogramming factors and p53 knockdown work together to induce activity by DNA demethylase proteins, thus promoting epigenetic remodeling of donor-cell and neuronal transcription networks during reprogramming.

An important consideration in developing patient-specific iN is the disease model's ability to accurately recapitulate disease phenotypes (Chanda et al., 2013). Disease modeling for neurological disorders, especially ones that are prevalent in the aging population, has been limited by the ability to fully recapitulate disease progression in neuronal cells. Direct reprogramming was one of the first steps to overcome limitations in studying age-associated phenotypes in neurons (Mertens et al., 2015; Huh et al., 2016; Liu et al., 2016; Tang et al., 2017). An extensive transcriptome analysis showed retention of age-related transcriptional signatures in directly reprogrammed neurons, while these signatures were reset in iPSCs and iPSCs-derived neurons (Mertens et al., 2015). There also appeared to be distinct differences in the gene expression profile of iN derived from young and old fibroblasts. *RanBP17*, a nuclear transport receptor, was decreased in human brain tissue samples from aged donors and corresponded to subsequent findings in iN cultures that the nuclear pore weakens with age, thereby effecting *RanBP17*-mediated transport of nuclear proteins in mature iN. A separate study confirmed the retention of cellular aging phenotypes by directly reprogramming young and aged fibroblasts to medium spiny neurons, which had comparable DNA methylation patterns to their donor cell (Huh et al., 2016). These findings suggest a key biomarker for age is preserved during the reprogramming process. However, age-related phenotypes in iN are not fully elucidated as some groups have discovered that iN have a similar transcriptome to fetal neurons and can be characterized electrophysiologically as immature, transitional, or highly functional. In addition, culturing neurons for extensive periods, with or without growth factors and glial co-cultures scarcely enhances the functional characteristics (Bardy et al., 2016; Lim et al., 2017). This inability to recapitulate a critical neuronal property is also observed with iPSC-derived neurons and has become a challenge with *in vitro* disease modeling as it is difficult to determine if functional dysregulation results from inefficient reprogramming strategies or disease pathology. Some groups have highlighted this constraint as a feature that may make some reprogramming methods more applicable for studying genetic pre-disposition and neurodevelopmental disorders (Marchetto et al., 2010; Brennand, 2013).

Progress has been achieved with artificially inducing aged phenotypes in neuronal models of Parkinson's disease by manipulating progerin expression and telomerase activity (Miller et al., 2013; Vera et al., 2016). Progerin is associated with Hutchinson-Gilford progeria syndrome (HGPS), a genetic disorder that results in pre-mature aging (Miller et al., 2013). Progerin overexpression in iPSC-derived dopaminergic neurons induced age-associated phenotypes, such as accumulation of neuromelanin (Miller et al., 2013). Telomere length has been used to determine if iPSC-derived neurons and directly reprogrammed neurons retain the age signature of the donor cell. Telomerase activity declines postnatally, resulting in telomere shortening as cells continue to undergo divisions. Inhibiting telomerase activity in iPSCs recapitulated age-associated phenotypes such as DNA damage and reactive oxygen species levels following differentiation to dopaminergic neurons (Vera et al., 2016). While these studies have primarily been validated in iPSC-derived neurons, the results suggest that artificially inducing age *in vitro* is possible. Subsequent studies will need to evaluate if these methods are applicable for artificially inducing age in iN and disease-relevant neuronal models.

## Direct Reprogramming With DNA Binding Domains

**Figure 3:**
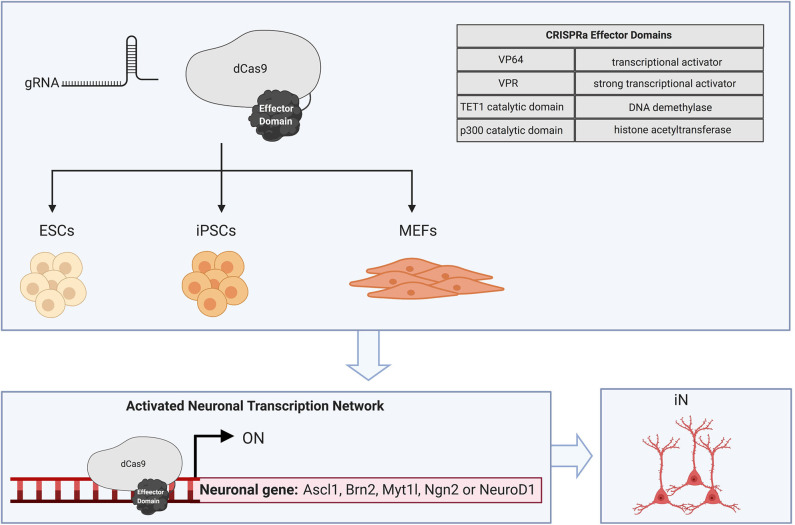
CRISPRa-mediated cellular reprogramming. CRISPR dCas9 fused to effector domains can be directed to specific endogenous genomic loci with a site-specific gRNA. Cellular reprogramming to the neuronal fate has been achieved in ESCs, iPSCs, and MEFs by targeting dCas9 activators to the putative transcription start site which promotes recruitment of chromatin remodeling factors and activating histone modifications. CRISPRa of neurogenic factors such as Ascl1, Brn2, Myt1l, Ngn2, and NeuroD1 is sufficient to create induced neurons.

Direct reprogramming can now be achieved by using programmable DNA binding domains to activate endogenous genes and modify the epigenetic landscape (Chakraborty et al., 2014; Black et al., 2016; Baumann et al., 2019) (Figure 3). Zinc finger proteins (*Zfp*) are a class of transcriptional activators with a DNA binding domain that can be targeted to neuronal genes (Liu et al., 2002). Zinc finger protein 521 (*Zfp521)* facilitates the conversion of mouse fibroblasts and human neonatal, fetal, or adult fibroblasts to neural stem cells with the potential to differentiate into neural progenitor and glial cells. The reprogramming timeline with transcriptional activators appears to be similar to traditional methods, as *Zfp521* needed to have activity for 24 days in order for the NSC transcriptome to be established. Interestingly, the addition of a small molecule cocktail was necessary to reprogram adult dermal fibroblasts, suggesting *Zfp521* alone is unable to overcome transcriptional and cell-fate barriers (Shahbazi et al., 2016).

The clustered regularly interspaced short palindromic repeats (CRISPR) and CRISPR-associated protein (Cas9) technology has been adapted for endogenous gene regulation and epigenetic editing. A nuclease deficient Cas9 (dCas9) which retains its DNA binding affinity can be engineered by inactivating the RuvC and HNH catalytic domains. These dCas9 complexes are directed to genomic regions that are complementary to a guide RNA (gRNA) sequence (Jinek et al., 2012). dCas9-mediated activation appears to be dependent on the chromatin context of the target region, guide RNA (gRNA) binding sites and an appropriate activator domain (Maeder et al., 2013b; Chakraborty et al., 2014; Baumann et al., 2019). Tailoring gRNA to bind in regulatory regions, such as enhancers and promoters can be an efficient approach for driving robust activation and modulating expression of transcriptional networks (Baumann et al., 2019; Matharu et al., 2019). Interestingly, co-delivery of multiple gRNA appears to produce a synergistic effect on activation at many genomic sites (Cheng et al., 2013; Maeder et al., 2013b; Black et al., 2016; Liu P. et al., 2018; Baumann et al., 2019; Savell et al., 2019).

CRISPR activators (CRISPRa) have also been employed to facilitate tunable regulation from neuronal promoters in primary rat neurons (Savell et al., 2019). The tunable expression achieved with artificial transcriptional activators, can be used to regulate the amount of gene activation required to drive reprogramming, an aspect that is largely inaccessible with traditional reprogramming methods. Moreover, interactions between the endogenous genes driving a particular cell-fate can be elucidated with CRISPRa and subsequently targeted to facilitate reprogramming (Perez-Pinera et al., 2013; Black et al., 2016; Liu Y. et al., 2018; Savell et al., 2019) A screen for factors involved in reprogramming to the neuronal cell fate was achieved with CRISPRa. This study differentiated mouse ESCs to neurons with an individual gRNA targeting endogenous *Ascl1* or *Ngn2*. Notably, gene interaction maps in these studies uncovered a novel role for the histone methyltransferase, Ezh2 which is the catalytic subunit of the polycomb repressive complex 2 (PRC2). RNA-seq results suggest Ezh2 facilitates differentiation of mouse ESCs and reprogramming of fibroblasts to neurons by repressing endodermal and mesodermal cell-fate determining genes (Liu Y. et al., 2018). These findings highlight the advantages of targeting endogenous neuronal networks and how transcription factor or epigenetic regulator interactions can be modulated to recapitulate the epigenomic landscape during neurodevelopment.

The binding of dCas9 activators near the putative transcription start site can promote recruitment of chromatin remodeling factors and activating histone modifications to DNA sequences within the promoter (Perez-Pinera et al., 2013; Qi et al., 2013; Chavez et al., 2015; Black et al., 2016; Thakore et al., 2016). Some groups that have modulated gene activity with artificial transcriptional activators rely on tiling gRNA binding sites to promote recruitment of multiple dCas9 activators. These approaches were applied for endogenous gene activation of the BAM factors in MEFs (Black et al., 2016). Here, direct reprograming of MEFs was initiated by fusing the VP16 tetramer, VP64 to the N and C terminus of dCas9 and tiling gRNA binding sites to BAM regulatory regions. However, the addition of small molecules to the neuronal culture medium was required to reprogram MEFs to neuronal cells positive for Tuj1 and Map2. Notably, enrichment of histone marks associated with active transcription occurred 3 days post-transfection at the endogenous BAM loci. These findings suggest that VP64-dCas9-VP64 which recruits chromatin remodeling factors, can indirectly alter the epigenetic landscape at target neuronal cell-fate determining genes. Robust gene expression with VP64 has also been achieved with the Suntag polypeptide chain, where VP64 is recruited to a repeating scaffolding sequence thereby enabling multiple VP64 activator domains to fuse to a single dCas9 protein (Tanenbaum et al., 2014). dCas9-Suntag achieved robust activation at endogenous *Ascl1* and *Ngn2* (Tanenbaum et al., 2014; Liu Y. et al., 2018). This method for recruiting multiple VP64 activator domains to enhancer and promoter regions reprogrammed MEFs to iPSCs and differentiated mouse ESCs to neurons (Liu P. et al., 2018; Liu Y. et al., 2018). Non-neuronal genes, such as *Myod1*, targeted with VP64-dCas9-VP64 induce reprogramming to skeletal myocytes and reveal a persistent activation of *Myod1* after dCas9 activation machinery has subsided (Chakraborty et al., 2014).

Recent studies have shown promising results targeting endogenous genes with alternative transcriptional activators, such as VP64, p65, and Rta (VPR) or epigenetic modifier domains, including catalytic domains of the histone acetyltransferase p300 and DNA demethylase TET1 (Perez-Pinera et al., 2013; Chavez et al., 2015; Black et al., 2016; Baumann et al., 2019; Halmai et al., 2020). Successful endogenous gene activation was achieved by creating a hybrid transcriptional activator domain VPR (Chavez et al., 2015; Thakore et al., 2016). The VPR-fused domain outperformed individual VP64, p65, or Rta domains as well as other combinations of the hybrid activator. The VPR fusion targeted to NEUROG2 or NEUROD1 successfully differentiated iPSCs to neuronal cells (Chavez et al., 2015). Epigenetic effector domains, such as p300 and TET1 have the potential to overcome barriers to reprogramming to the neuronal lineage by directly rewriting the epigenetics of the intended target genes (Baumann et al., 2019). Endogenous gene expression can be epigenetically altered by the DNA demethylation activity of TET1 (Maeder et al., 2013a). dCas9-TET1 is sufficient to remove transcriptional barriers, such as CpG island methylation, in the *Sox1* promoter region and initiate reprogramming to a NSC state. Interestingly, this reprogramming was dependent on co-delivery of the dCas9-VP64 activator and dCas9-TET1 epigenetic editor, suggesting different artificial transcriptional activators may be required to edit the epigenetic landscape at certain genomic loci (Baumann et al., 2019). Acetylation of H3K27 in enhancer regions can be achieved with a single gRNA and dCas9 fusion to the p300 acetyltransferase (Hilton et al., 2015). This epigenetic editor domain may be effective at facilitating reprogramming, as p300 can be used to activate neuronal cell-fate determining genes from proximal or distal enhancers.

## Conclusion

Here, we have reviewed the various methods employed to directly reprogramming somatic cells to iN. Most research to date has focused on using exogenous reprogramming factors, microRNAs, or small molecules to directly reprogram somatic cells to neuronal lineages (Table 1). However, the mechanisms underlying reprogramming are diverse and comprehensive analysis is required to determine if current methods are sufficient to recapitulate the epigenomic and transcriptomic signatures observed during neurogenesis. Additional research is necessary to determine which distinct transcriptional networks are associated with neuronal subtypes and the reprogramming strategies sufficient to induce the diverse neuronal identities present in the human brain. iN conversion with transcription factors has shown the importance of employing neuronal-cell fate master regulators in addition to factors promoting neuronal maturation. However, reprogramming to iN subtypes likely requires additional reprogramming factors to specify neuronal identity (Tsunemoto et al., 2018). It is also unclear which reprogramming methods enable the timing of neuronal subtype specification to be modeled *in vitro*. A recent report showed combined expression of miRNA 9/9^*^, miRNA 124, ISL1, and LHX3 was sufficient to create motor neurons. Interestingly, this study subsequently described how microRNA 9/9^*^ and miRNA 124 induction created global changes to the epigenomic landscape through altered expression of DNA methyltransferases, histone, and chromatin remodelers (Abernathy et al., 2017). Likewise, motor iN were created by pairing small molecules for neuronal pathway activation with the master regulator *NGN2*, in addition to the motor neuron factors, *ISL1* and *LHX* (Liu et al., 2016). These studies suggest neuronal subtype conversion may rely on targeting different transcriptional networks with multiple reprogramming methods. Moreover, optimizing strategies to create specific neuronal subtypes affected in neurodegenerative disorders is essential to further understand disease pathology and subsequently employ techniques to artificially induce relevant phenotypes. These studies will be critical to developing accurate disease-in-a-dish models for neurodegenerative disorders.

As the reprogramming tool kit continues to develop, it is now possible to reprogram with approaches which activate endogenous neuronal-cell fate determining genes. The CRISPR reprogramming technology is poised to enable coordinated expression of endogenous neuronal networks when coupled with activator domains that catalyze DNA demethylation or histone acetylation. Leveraging these targeted approaches to influence gene expression at the chromatin level has the potential to overcome barriers to reprogramming and accelerate the development of neuronal disease models for broad applications. Chromatin immunoprecipitation sequencing (ChIP-seq) showed rapid modifications to chromatin accessibility within 48 h of transducing MEFs with Ascl1 (Wapinski et al., 2017). Whether ASCL1 induces similar changes to chromatin state when its endogenous locus is targeted in human fibroblasts, remains unclear. As these reprogramming strategies continue to be refined, it is necessary for these techniques to be employed with human somatic cells, to advance disease modeling efforts and the validation of potential treatments for human neurodegenerative disorders. A majority of the information on inducing the neuronal cell-fate has been derived from studies from murine neurodevelopment or derived cells. Employing these distinct reprogramming strategies in human somatic cells is also necessary to increase reprogramming efficiency which is significantly lower compared to mouse-derived iN (Chanda et al., 2014). Indeed, a major advantage to reprogramming cell identity with CRISPR/dCas9 fused to effector domains or epigenetic writers and erasers, is the efficiency in which gRNA can be designed to activate target regions. The ability to concurrently activate neuronal-fate determining genes with respective gRNA presents a novel platform to achieve physiologically relevant gene expression. There are a multitude of reprogramming techniques, yet it is becoming increasingly evident that extensive characterization is essential to characterize iN. In addition to assessing morphology and functional properties, changes in DNA methylation patterns, histone modifications, and the transcriptome can pinpoint reprogramming milestones as the donor cell transcriptome is repressed. Moreover, these studies benefit groups developing disease models as incorporating studies on DNA methylation provides insight into age-related signatures. Maturation is largely stunted *in vitro*; however, recent advances in artificially inducing age show *in vitro* models can be improved to model late-onset neurodegenerative disorders (Miller et al., 2013; Vera et al., 2016; Lim et al., 2017; Mehta et al., 2018). These advances in iN reprogramming and disease modeling will continue to contribute to uncovering disease variations and advancing personalized medicine for individuals with neurodegenerative disorders.

## Author Contributions

JC designed the review. JC, JH, and KF contributed to the conceptualization of the review. JC wrote the paper with input from all authors. KF edited and approved the final manuscript. All authors contributed to the article and approved the submitted version.

## Conflict of Interest

The authors declare that the research was conducted in the absence of any commercial or financial relationships that could be construed as a potential conflict of interest.
